# The role of left atrial strain in patients with functional tricuspid regurgitation before and after annuloplasty: a long-term follow-up study

**DOI:** 10.1186/s12947-021-00264-z

**Published:** 2021-10-18

**Authors:** Qing-long Meng, Hong Meng, Jia Tao, Shu Yang, Hao Wang

**Affiliations:** 1 Department of Echocardiography, Fuwai Hospital, National Center for Cardiovascular Diseases, Chinese Academy of Medical Sciences and Peking Union Medical College, 167 Beilishi Road, XiCheng District, Beijing, 100037 People’s Republic of China; 2 Philips (China) Investment Co., Ltd, Shenyang, 110000 People’s Republic of China

**Keywords:** Left atrial strain, Functional tricuspid regurgitation, Recurrence

## Abstract

**Background:**

Functional tricuspid regurgitation (TR) is common among patients with left heart disease and may recur during the follow-up period after selective tricuspid valve annuloplasty (TVA). This study aims to analyse the relationship between left atrial (LA) strain and the degree of preoperative functional TR and to explore the role of LA strain in predicting TR recurrence.

**Methods:**

This study included 63 patients with rheumatic mitral stenosis who underwent mitral valve replacement and concomitant TVA. Additionally, 20 healthy controls were enrolled. Preoperative conventional LA echocardiographic parameters and LA strain were measured. The association between LA strain and preoperative functional TR severity was analysed by Pearson correlation. Predictors of recurrent TR were determined by multivariate logistic regression analyses.

**Results:**

Compared with the control group, the mitral stenosis group developed a significant impairment in terms of LA strain. The degree of preoperative functional TR exhibited moderate correlations with LA reservoir strain (*r* = − 0.57) and LA conduit strain (*r* = 0.48). During a median follow-up period of 66.4 ± 36.4 months, TR recurred in 18 patients. Preoperative LA reservoir strain and the mean transmitral gradient were predictors of postoperative TR recurrence. When the two indexes were combined to establish a prediction, the sensitivity and specificity of prediction increased. The area under the receiver operating characteristic curve of the combined indicator was higher than those of the single indicators (0.90 vs. 0.70 and 0.72).

**Conclusions:**

LA strain correlates with preoperative functional TR severity in patients with rheumatic mitral stenosis. The LA reservoir strain and preoperative mean transmitral gradient are independent predictive factors for recurrent TR after TVA.

**Supplementary Information:**

The online version contains supplementary material available at 10.1186/s12947-021-00264-z.

## Introduction

Functional tricuspid regurgitation (TR) is quite common in left heart disease, and several studies have confirmed that preoperative functional TR may progress if left untreated at the time of left-sided valve surgery. Selective tricuspid valve annuloplasty (TVA) can prevent TR from worsening and thus decrease both postoperative mortality and morbidity. However, even after the appropriate intervention, moderate-to-severe TR recurs in up to 15 to 20% of patients within the first year and in 30 to 70% of patients within 3–5 years and is closely related to increased mortality [1]. Previous studies have reported predictors of TR recurrence after annuloplasty, including right ventricular (RV) dysfunction, pulmonary hypertension, and preoperative tricuspid valve annular diameter. Interestingly, however, left atrial (LA) dysfunction has rarely been investigated in these situations. Park and colleagues reported that improvement of LA function after the maze procedure was strongly associated with a significantly decreased risk of late progression of TR [2]. Kim et al. also confirmed that the maintenance and recovery of LA mechanical function are valuable for preventing progression of functional TR in patients undergoing left-sided valve surgery [3]. However, these studies were mainly concerned with the effects of conventional echocardiographic parameters on functional TR, which have limited value in reflecting LA function. Several studies have noted that the LA strain, particularly reservoir strain, is crucial for non-invasive function assessment and may be valuable for clinical evaluation and earlier therapeutic intervention [4, 5]. The role of LA strain in patients with functional TR before and after TVA is still unknown. In this study, LA function was suggested to play crucial roles in the onset, development, and progression of functional TR. Whether combining preoperative LA strain with traditional echocardiographic indicators could be a better non-invasive tool for predicting TR recurrence also needs to be further investigated.

## Methods

### Study population

From March 2011 to January 2015, we initially enrolled 67 patients with rheumatic mitral stenosis who underwent complete echocardiography assessment before mitral valve replacement (MVR) with concomitant TVA for functional TR. The presence of mitral leaflet rheumatic disease was determined by pathological findings. The exclusion criteria included the presence of tricuspid leaflet rheumatic disease determined either by preoperative echocardiography or by surgeon evaluation during surgery, pacemaker wires across the tricuspid valve preoperatively, congenital valvular disease, severe mitral regurgitation, and poor image quality. The indication for TVA was mild to severe functional TR with or without a systolic tricuspid annular diameter > 20 mm/m^2^ [6]. All patients underwent surgery through a median sternotomy. The annuloplasty type depended on the operator’s preference. Modified DeVega suture annuloplasty was performed in 39 (58.2%) patients, and ring annuloplasty (Duran, Medtronic, Inc., Minneapolis, MN) was performed in 28 (41.8%) patients. Surgical procedures may have included the Cox Maze procedure and/or aortic valve replacement. At the same time, 20 healthy volunteers with similar age distributions were enrolled to serve as the control group. This retrospective study was approved by the hospital ethics committee, and informed consent was waived.

### Echocardiography analyses

Preoperative transthoracic echocardiography (TTE) was obtained while the patients were at rest within 2 weeks before surgery using a Philips IE33 ultrasound device (Philips Medical Systems, Andover, MA, USA). Postoperative echocardiographic follow-up was performed prior to discharge. Further follow-up echocardiography was performed according to the recommendation of the patient’s attending physician. All patients underwent 3 or more echocardiographic assessments and clinical visits after discharge. Two-dimensional echocardiographic measurements and Doppler evaluation were performed according to the American Society of Echocardiography guidelines [7]. Five cardiac cycles were collected for analysis in atrial fibrillation patients, and three cardiac cycles were collected in sinus rhythm patients.

The mitral valve area was evaluated by 2-dimensional planimetry (MVA-2D), the pressure half-time method (MVA-PHT), and the velocity-time integral of the mitral valve for the peak and mean transmitral gradient [8]. The degree of TR was evaluated by a multiparametric approach, including an assessment of the colour Doppler–derived jet area, the vena contracta width, the continuous-wave Doppler–derived jet density and contour, and the hepatic vein flow velocity pattern; on this basis, TR was graded as mild (1+), mild to moderate (1.5+), moderate (2+), moderate to severe (2.5+), or severe (3+) [9]. The peak early diastolic velocity (E) of the tricuspid valve was measured by pulsed Doppler echocardiography from the apical four-chamber view. The peak early diastolic myocardial velocity (E′) and peak systolic myocardial velocity (S′) were obtained by Doppler tissue imaging at the levels of the basal portion of the anterior tricuspid annulus. The maximum (end-diastole) and minimum (end-systole) tricuspid annular (TA) diameter was measured from the apical four-chamber view. Pulmonary artery systolic pressure (PASP) was estimated from the tricuspid regurgitant jet velocity and right atrium pressure in accordance with the modified Bernoulli equation. The left ventricular end-systolic volume, end-diastolic volume, and ejection fraction were assessed by the biplane Simpson method. The following volumetric parameters of the left atrium were also evaluated by the Simpson method: (1) maximum LA volume at end-systole (LAV max); (2) maximum LA volume index (LAVI), calculated by dividing the LAV max by body surface area; (3) minimum LA volume at end-diastole (LAV min); (4) LA stroke volume (LASV) = LAV max−LAV min; (5) LA emptying fraction (LAEF) = (LA max−LA min)/LA max × 100; and (6) LA expansion index (LAEI) = (LA max−LA min)/LA min × 100.

**Fig. 1:**
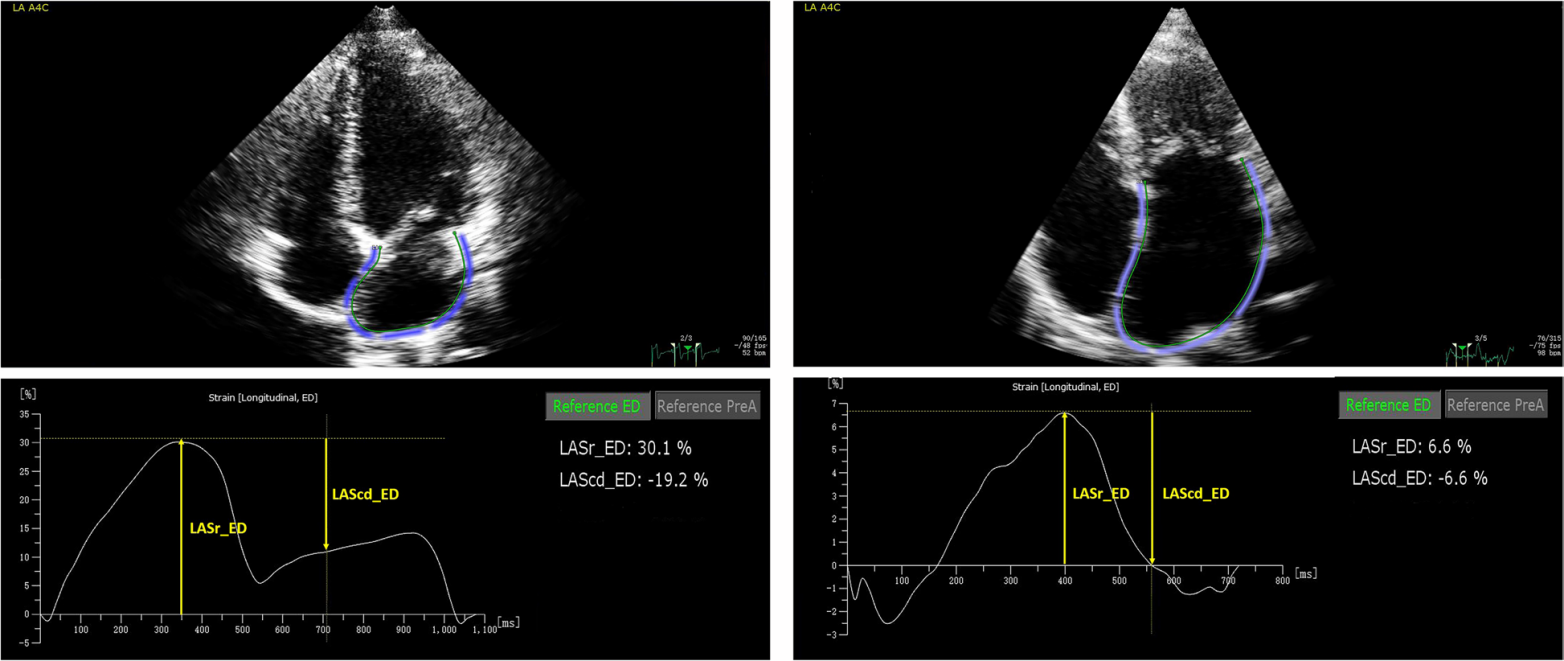
The figure presents the strain of the left atrium using two-dimensional speckle-tracking echocardiography in patients with sinus rhythm (**A**, **B**) and atrial fibrillation (**C**, **D**). AutoStrain LA software will automatically detect and track the contours and then advance to the analysis step. Left atrial reservoir strain and left atrial conduit strain are displayed based on the end-diastolic option

Two-dimensional speckle-tracking echocardiography (2D-STE) was analysed offline using QLAB AutoStrain LA software (Philips Medical Systems). Two-dimensional echocardiographic images collected from the non-foreshortened apical four-chamber view were acquired for further analysis. The atrial appendage and pulmonary veins were excluded from the LA cavity [10]. First, the software can automatically identify which selected image is an apical four-chamber view. Then, the observer manually traced the endocardial LA border in the four-chamber view in end diastole and the software automatically track speckles along the endocardial border throughout the selected cardiac cycle. Researchers can retrace any segments not tracked in any part of the endocardial LA wall to obtain satisfactory results. The frame rate was 60–65 Hz. The reference point for zero strain was set at LV end-diastole following mitral valve closure. The LA strain is the difference in the measurements of the phases. In patients with sinus rhythm, LA reservoir strain is measured as difference in the strain value between mitral valve opening and LV end-diastole (positive value), and LA conduit strain is measured as the difference in the strain value between the onset of atrial contraction and mitral valve opening (negative value). In patients with atrial fibrillation, LA reservoir strain was obtained using the same method, and LA conduit strain has the same value as LA reservoir strain, but with a negative sign (Fig. 1). The left ventricular global longitudinal strain was measured by AutoStrain LV software with QLAB using the three standard apical views (four-chamber, two-chamber, and three-chamber views). All echocardiographic values were measured three times by the same operator based on a single-cycle image, and the results shown were averaged. In the present study, we evaluated the inter- and intra-observer variabilities of LA strain measurements in a randomly selected subset of 20 patients. The LA strain parameters were analysed twice by one investigator. The second investigator was blinded to the initial results.

### Statistical analysis

A Kolmogorov-Smirnov test was used to determine whether the data satisfied the normal distribution criteria. Quantitative values are expressed as the mean ± SD, and categorical variables are represented as absolute numbers or proportions. An independent *t*-test was used to compare continuous values between the two groups for normally distributed variables. Categorical variables were compared using the χ^2^ test or Fisher’s exact test. Pearson’s correlation was used to analyse associations between LA echocardiographic and strain parameters and the degree of preoperative functional TR. We conducted a univariate analysis to identify all variables that were significantly related to the recurrence of TR in the study. Multivariate analysis with logistic regression was conducted with the recurrence of TR as the dependent variable. The cutoff value was determined from the area under the receiver operating characteristic (ROC) curve (AUC) to provide the best sensitivity and specificity for each parameter for predicting recurrent TR. Interobserver and intraobserver variability for measurements of LA strain were analysed by Bland–Altman plots. Differences were considered significant at *p* < 0.05. All analyses were performed using SPSS 24.0 (IBM Corp., Armonk, NJ, USA), MedCalc 18.2.1 (MedCalc Software, Ltd., Ostend, Belgium), and GraphPad Prism 8.0.2 (GraphPad Software, San Diego, CA, USA).

## Results

### Baseline patient characteristics

A total of 67 patients with rheumatic mitral stenosis and different degrees of functional TR who received complete clinical and echocardiographic examinations were initially included. Four patients were lost to follow-up. The final cohort of this study consisted of 63 patients (22 men, 41 women; mean age 52.6 ± 8.2 years), whose mean follow-up period was 66.4 ± 36.4 months, with the longest follow-up duration being 111 months. The demographic features and preoperative clinical characteristics of patients and healthy volunteers are presented in Table 1. Among the patients, three had hypertension, 51 had atrial fibrillation, and 24 were classified as New York Heart Association (NYHA) classes III to IV. The mitral stenosis group had higher heart rates than the control group. Other baseline characteristics showed no differences between the two groups. Postoperative echocardiography performed prior to discharge showed a TR severity < 1+ for all patients. Successful TVA was defined as TR severity < 2+ during the follow-up period after surgery (Group I, *n* = 45). TR severity ≥2+ was considered recurrence (Group II, *n* = 18). No significant differences were found between Group I and Group II in terms of age, weight, height, body surface area, or other medical histories.

**Table 1:** Demographic features, preoperative clinical characteristics and echocardiographic variables of patients

	Control (*n* = 20)	Patients with MS and functional TR (*n* = 63)	Patients undergoing TVA		*P* value, Control vs. functional TR	*P* value, Group I vs. Group II
			Group I (*n* = 45)	Group II (*n* = 18)		
Demographic features						
Age (years)	48.4 ± 9.0	52.6 ± 8.2	52.4 ± 7.4	53.1 ± 10.2	0.06	0.77
Sex (M/F)	13/7	22/41	12/33	10/8	0.02	0.04
Weight (kg)	68.8 ± 11.5	63.4 ± 9.9	63.8 ± 10.6	62.2 ± 8.1	0.07	0.55
Height (cm)	169.9 ± 7.6	166.0 ± 7.8	165.5 ± 7.5	167.2 ± 8.7	0.05	0.44
BSA (m^2^)	1.79 ± 0.17	1.70 ± 0.16	1.70 ± 0.16	1.70 ± 0.15	0.06	0.92
Heart rate (beats/min)	71.0 ± 10.0	80.0 ± 21.0	76.0 ± 15.3	92.1 ± 28.4	0.00	0.03
Preoperative clinical characteristics						
NYHA functional class III-IV	–	24 (38.1%)	20 (44.4%)	4 (22.2%)	–	0.15
Hypertension	–	3 (0.05%)	1 (2.2%)	2 (11.1%)	–	0.19
Atrial fibrillation	–	51 (80.9%)	35 (77.8%)	16 (88.9%)	–	0.48
TR severity						
1+	–	19 (30.0%)	17 (37.8%)	2 (11.1%)	–	–
1.5+	–	16 (25.4%)	10 (22.2%)	6 (33.3%)	–	–
2+	–	14 (22.2%)	7 (15.6%)	7 (38.9%)	–	–
2.5+	–	3 (4.8%)	2 (4.4%)	1 (5.6%)	–	–
3+	–	11 (17.5%)	9 (20%)	2 (11.1%)	–	–
TR VCW (mm)	–	4.24 ± 2.33	4.11 ± 2.51	4.66 ± 1.87	–	0.41
Suture annuloplasty/Ring annuloplasty	–	36/27	23/22	13/5	–	0.13
Conventional echocardiographic variables						
MVA by 2D planimetry (cm^2^)	–	0.88 ± 0.15	1.10 ± 0.39	0.82 ± 0.29	–	0.01
MVA by PHT (cm^2^)	–	0.92 ± 0.38	1.11 ± 0.10	0.86 ± 0.22	–	0.04
MV mean gradient (mm Hg)	–	10.0 ± 4.8	8.9 ± 4.0	12.7 ± 5.7	–	0.00
MV peak gradient (mm Hg)	–	19.4 ± 7.6	19.2 ± 7.9	19.9 ± 6.9	–	0.74
TA maximal diameter (cm)	3.39 ± 0.48	3.44 ± 0.46	3.45 ± 0.49	3.42 ± 0.37	0.00	0.83
TA minimal diameter (cm)	2.86 ± 0.31	3.39 ± 0.39	3.40 ± 0.51	3.37 ± 0.41	0.00	0.82
PASP (mm Hg)	–	44.7 ± 22.6	42.0 ± 22.4	51.4 ± 22.4	–	0.14
Tricuspid E (cm/s)	56.3 ± 7.6	63.4 ± 19.3	63.7 ± 20.0	62.9 ± 18.0	0.02	0.89
E’ (cm/s)	11.9 ± 2.2	11.4 ± 2.9	11.1 ± 2.7	11.9 ± 3.3	0.48	0.41
S′ (cm/s)	12.7 ± 2.0	12.1 ± 2.6	12.3 ± 2.7	11.8 ± 2.3	0.44	0.55
E/E’	4.8 ± 0.9	5.8 ± 2.5	5.9 ± 2.3	5.5 ± 2.8	0.06	0.63
LA-ap (mm)	32.4 ± 2.3	55.5 ± 12.0	55.9 ± 12.9	54.6 ± 9.4	0.00	0.71
LA maximal volume (mL)	38.0 ± 10.4	202.5 ± 27.3	228.0 ± 27.2	138.7 ± 15.2	0.00	0.03
LA minimal volume (mL)	12.5 ± 4.7	160.1 ± 23.8	182.4 ± 32.3	104.2 ± 14.4	0.00	0.03
LAVI (mL/m2)	21.2 ± 5.8	119.7 ± 15.8	134.9 ± 21.4	81.7 ± 8.8	0.00	0.02
LASV (mL)	25.5 ± 6.7	42.5 ± 4.3	45.6 ± 5.5	34.6 ± 5.2	0.00	0.24
LAEF (%)	67.5 ± 5.3	24.9 ± 11.9	24.4 ± 1.6	26.1 ± 3.5	0.00	0.60
LAEI (%)	216.0 ± 52.2	39.0 ± 44.1	34.9 ± 20.3	49.2 ± 18.1	0.00	0.25
LV end-diastolic volume (mL)	81.9 ± 20.9	87.9 ± 34.7	93.6 ± 38.2	73.7 ± 18.1	0.47	0.01
LV end-systolic volume (mL)	34.4 ± 11.0	41.7 ± 19.5	44.8 ± 21.2	33.9 ± 11.9	0.04	0.01
LV EF (%)	58.5 ± 4.7	53.0 ± 8.7	52.6 ± 7.9	53.9 ± 10.5	0.00	0.57
2D-STE variables						
LAS-r (%)	42.5 ± 10.2	7.6 ± 4.0	8.5 ± 4.1	5.4 ± 2.5	0.00	0.00
LAS-cd (%)	−25.9 ± 7.4	−6.8 ± 3.7	−7.5 ± 3.9	−5.1 ± 2.3	0.00	0.02
LV-PGLS (%)	−22.7 ± 3.5	−15.6 ± 4.9	−15.5 ± 4.4	−15.8 ± 6.1	0.00	0.86

*MS* Mitral stenosis, *TR* Tricuspid regurgitation, *TVA* Tricuspid valve annuloplasty, *BSA* Body surface area, *VCW* Vena contracta width, *MVA* Mitral valve area, *PHT* Pressure half-time, *TA* Tricuspid annular, *PASP* Pulmonary arterial systolic pressure, *LA-ap* Left atrial anterior-posterior diameter, *LAVI* Left atrial maximal volume index, *LASV* Left atrial stroke volume, *LAEF* Left atrial emptying fraction, *LAEI* Left atrial expansion index, *LVEF* Left ventricular ejection fraction, *2D-STE* Two-dimensional speckle-tracking echocardiography, *LAS-r* Left atrial reservoir strain, *LAS-cd* Left atrial conduit strain, *LV-PGLS* Left ventricular peak global longitudinal strain

During the follow-up period of this study, 1 patient in the overall population died of a cerebrovascular accident after MVR. No patients underwent re-operative tricuspid valve annuloplasty and repair. In addition, 2 patients experienced postoperative new-onset atrial fibrillation, and all received medication for controlling ventricular rate control. Among patients from Group II, except for the necessary anticoagulation treatments of aspirin and warfarin, no medicine (including diuretics) was given in 16 patients. However, there were 2 readmissions for severe heart failure symptoms during this period; both of these patients were given active drug therapy.

### Conventional and 2D-STE variables

Compared to the control group, the mitral stenosis group had significantly lower LAEF, LAEI, left ventricular ejection fraction (LVEF), and left ventricular peak global longitudinal strain and significantly larger tricuspid annular diameter, LA volume, and LAVI. Meanwhile, LA reservoir strain and LA conduit strain were impaired in the mitral stenosis group. Compared with Group I, Group II had a significantly reduced preoperative mitral valve area and lower preoperative LA volume, left ventricular volume, and LA reservoir strain. However, patients in Group II had a higher preoperative mean transmitral gradient (Table 1).

### Correlates of functional TR

**Fig. 2:**
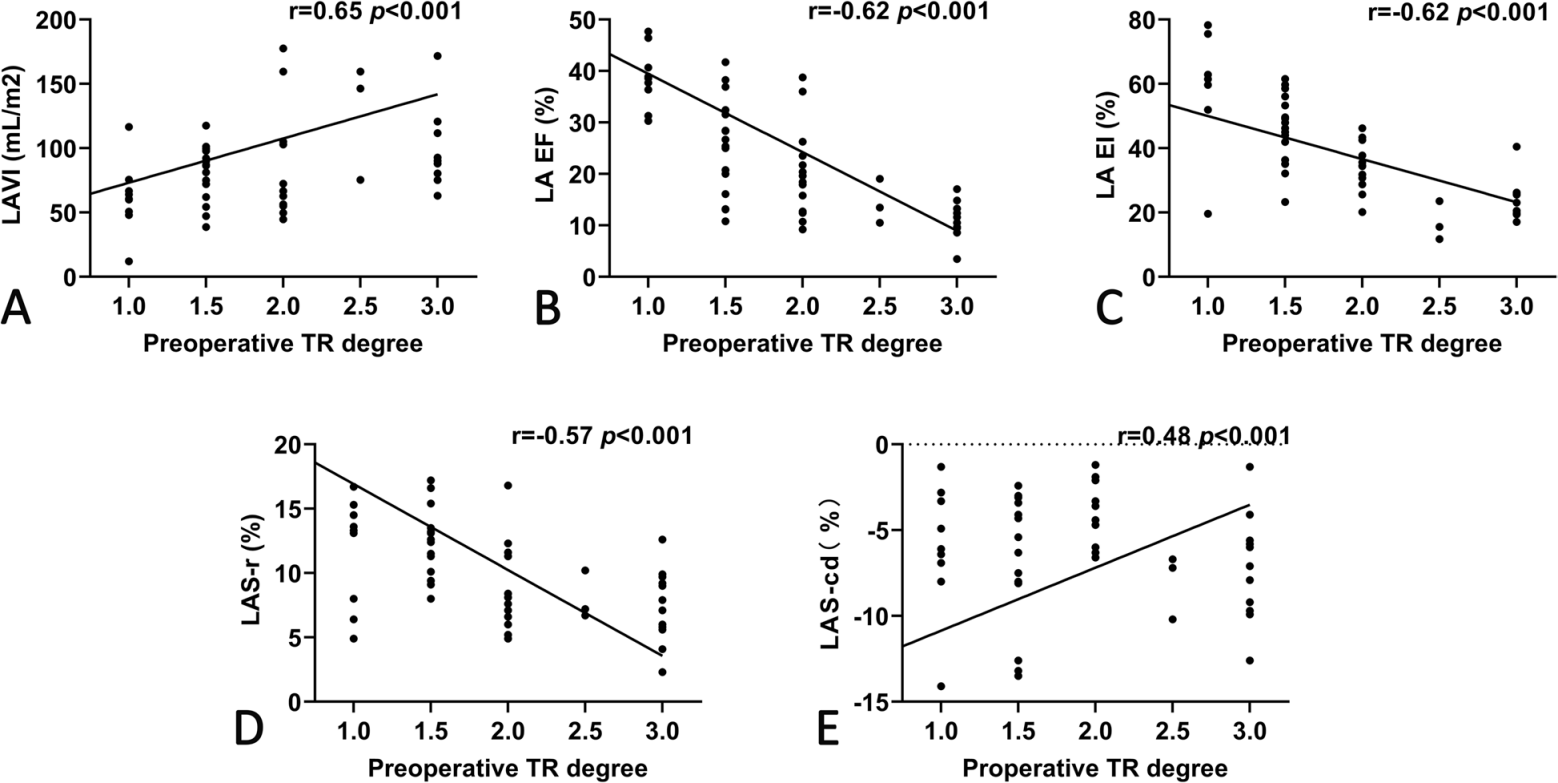
Regression plots showing the correlation of preoperative tricuspid regurgitation degree with several left atrial echocardiographic parameters. LAVI Left atrial maximal volume index; LAEF Left atrial emptying fraction; LAEI Left atrial expansion index; LAS-r Left atrial reservoir strain; LAS-cd Left atrial conduit strain; TR Tricuspid regurgitation

Figure 2 summarizes the associations between the degree of preoperative functional TR and echocardiographic indicators of the left atrium. For the entire cohort, the degree of functional TR correlated with the volumetric parameters of the left atrium, including LAVI, LA emptying fraction, and LA expansion index (*r* = 0.65, − 0.62, and − 0.62, respectively; *p* < 0.001 for all). At the same time, LA reservoir strain (*r* = − 0.57) and LA conduit strain (*r* = 0.48) showed moderate correlations with the degree of functional TR (*p* < 0.001 for both).

### Predictors of recurrent TR and ROC curve analysis

**Fig. 3:**
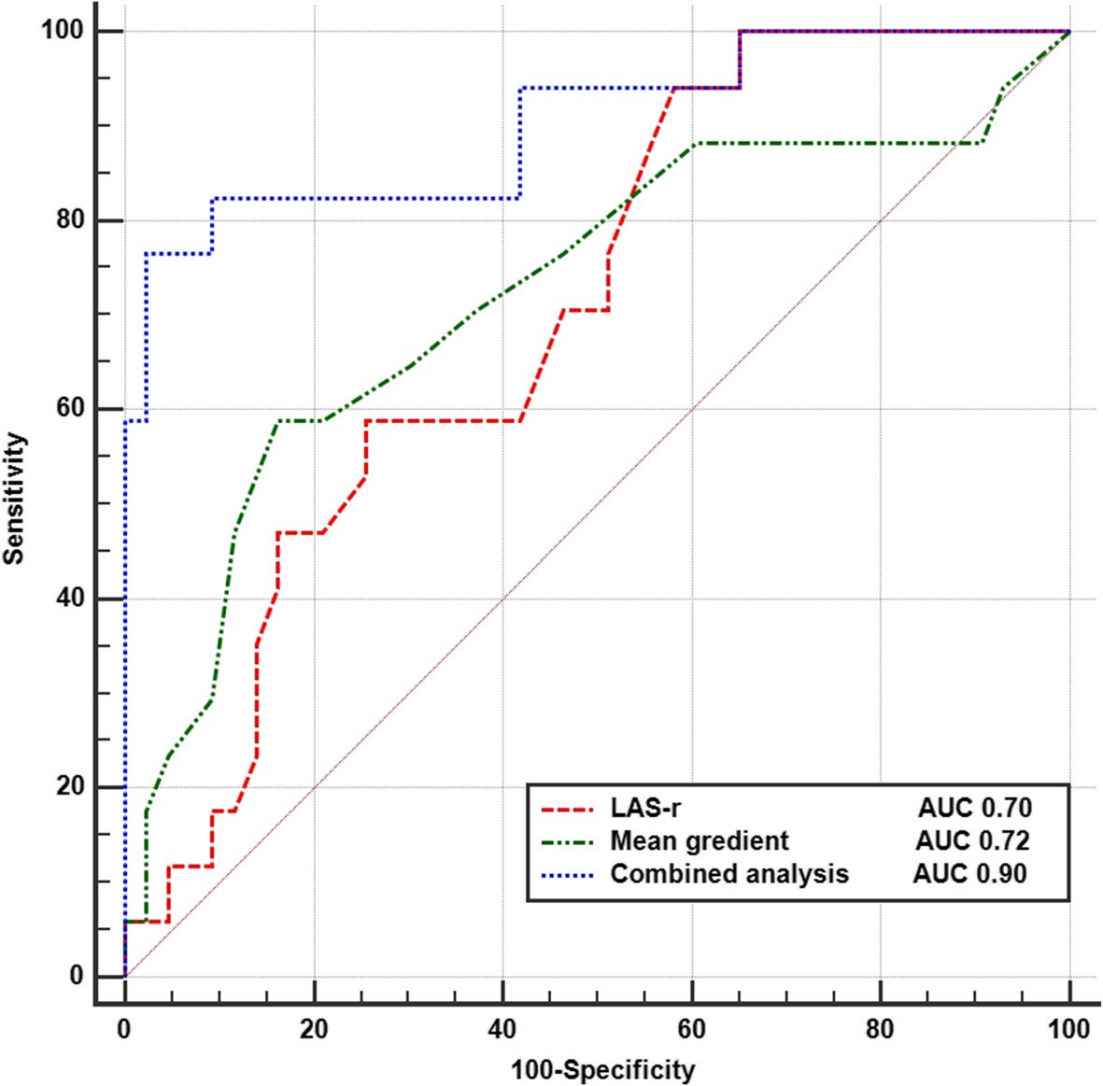
Receiver operating characteristic curve analyses of predictors for recurrence of functional tricuspid regurgitation. AUC area under the curve; LAS-r Left atrial reservoir strain

In the univariate logistic regression analysis, heart rate, preoperative mean transmitral gradient, and LA reservoir strain were correlated with recurrent TR. The MVA-2D, PASP, LV end-diastolic volume, LV end-systolic volume, LAV max, LAV min, LAVI, and LA conduit strain did not show significant predictive value in predicting TR recurrence. In the multivariate logistic regression analysis, preoperative LA reservoir strain (*p* = 0.04) and mean transmitral gradient (*p* = 0.02) were identified as independent predictors of recurrent TR (Table 2). After multivariate adjustment for TA maximal diameter, TA minimal diameter, and pre-operative TR severity, LA reservoir strain and mean transmitral gradient remained independent predictors (Supplementary Material Table S1). According to the ROC curve analysis, the sensitivities and specificities for predicting recurrent TR were 75.6 and 61.1% for LA reservoir strain < 5.9% (AUC: 0.70, 95% CI 0.59–0.85) and 55.6 and 83.7% for mean transmitral gradient > 12.5 mmHg (AUC: 0.72, 95% CI 0.57–0.87), respectively. When the preoperative LA reservoir strain and mean transmitral gradient were combined to predict recurrent TR, the combined indicators achieved the highest AUC (0.90, 95% CI 0.81–0.98), and its sensitivity and specificity were 76.6 and 97.7%, respectively (Fig. 3).

**Table 2:** Univariate and multivariate logistic regression analysis for predicting the recurrence of TR after TVA

	Univariable analysis			Multivariable analysis		
	β	95% CI	*P* value	β	95% CI	*P* value
Heart rate (beats/min)	1.04	1.01–1.07	0.01	1.02	0.99–1.06	0.18
Atrial fibrillation	0.44	0.09–2.23	0.32			
MVA by 2D planimetry (cm^2^)	0.07	0.01–0.59	0.07			
MV mean gradient (mm Hg)	1.19	1.04–1.35	0.01	1.29	1.04–1.60	0.02
PASP (mm Hg)	1.02	0.99–1.05	0.14			
LV end-diastolic volume (mL)	0.98	0.97–1.01	0.05			
LV end-systolic volume (mL)	0.96	0.93–1.01	0.05			
LA maximal volume (mL)	0.99	0.99–1.01	0.18			
LA minimal volume (mL)	0.99	0.99–1.01	0.18			
LAVI (mL/m2)	0.99	0.99–1.01	0.18			
LAS-r (%)	0.77	0.63–0.93	0.01	0.85	0.73–0.98	0.04
LAS-cd (%)	1.26	1.03–1.55	0.07			

*CI* Confidence interval, *MVA* Mitral valve area, *PASP P*ulmonary arterial systolic pressure, *LAVI* Left atrial maximal volume index, *LAS-r* Left atrial reservoir strain, *LAS-cd* Left atrial conduit strain

### Inter- and intraobserver variability for measurements

**Fig. 4:**
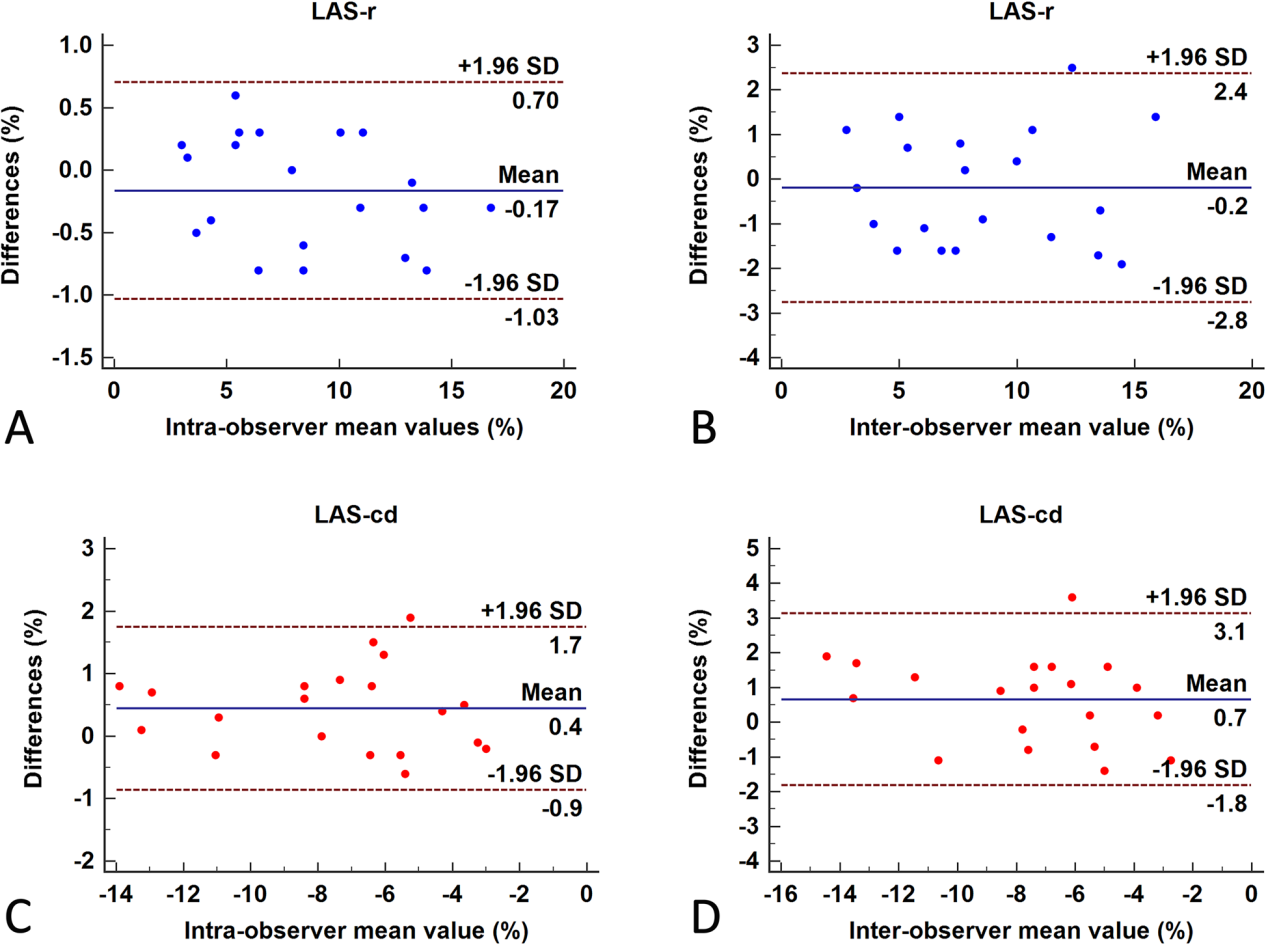
Bland–Altman plots of inter- and intra-observer variability. LAS-r Left atrial reservoir strain; LAS-cd Left atrial conduit strain

Bland–Altman plots of the interobserver and intraobserver variabilities for measurements of LA strain using 2D-STE are summarized in Fig. 4. The intraobserver and interobserver variabilities were within acceptable limits. The intraobserver intraclass correlation coefficients for LA reservoir strain and LA conduit strain were 0.97 and 0.97, respectively (*p* < 0.05 for both). Conversely, the interobserver intraclass correlation coefficients for LA reservoir strain and LA conduit strain were 0.94 and 0.93, respectively (*p* < 0.05 for both).

## Discussion

The present study had two major findings. First, preoperative functional TR severity was correlated with LA strain in rheumatic mitral stenosis patients; this is the first study to provide clear evidence of this association. Second, the preoperative LA reservoir strain and mean transmitral gradient were independent predictors of recurrent TR after TVA over long follow-up periods. Additionally, the AUC for the combination of the two indicators was 0.90, indicating that the combined indicators were more accurate than single indicators in predicting TR recurrence.

LA inflammation and fibrosis can be attributed to rheumatic carditis and lead to LA pressure overload. It is well known that these pathological and hemodynamic changes induce electrical and mechanical remodelling of the atrium and are closely related to subsequently increased pulmonary artery pressure, tricuspid annular dilatation, and functional TR development. Some researchers have shown that patients with rheumatic mitral stenosis are at increased risk of significant LA enlargement and dysfunction, and TR is relatively common (up to 55 to 60%) in these patients [11, 12]. Conversely, RV volume overload due to moderate-to-severe TR can result in left heart geometric alterations that reduce LA preload, impair left ventricular filling function, and decrease the atrial contribution to left ventricular filling [13, 14]. Thus, certain correlations between functional TR severity and LA function may exist. The present study demonstrates, on the one hand, that LA mechanics, including reservoir and conduit function, was markedly impaired in patients with rheumatic mitral stenosis. On the other hand, preoperative functional TR severity was found to correlate with LA strain in this study. The result suggests that LA functional changes are closely related to functional TR and supports our a priori hypothesis. A recent study in a population with severe pulmonary arterial hypertension also demonstrated decreased LA global strain and suggested that this reflects LA dysfunction in these patients [15]. Moreover, Ashwin et al. noted the exceptional value of LA strain in the diagnosis and treatment of pulmonary hypertension in their latest study [16]. All these findings indicate that LA strain has specific value in the diagnosis and assessment of right heart diseases; further research regarding the interrelationships among the right ventricle, pulmonary circulation, and left atrium is still needed.

TVA is a common strategy used for the treatment of significant functional TR, although reports of TR recurrence after the procedure exist. A 30 to 70% recurrence rate within 3 to 5 years can be reported even in the absence of organic changes, including infective endocarditis, degenerative changes, and rheumatic damage, and recurrence is associated with a poor prognosis [17, 18]. Therefore, relatively accurate predictors of TR recurrence are needed and are expected to provide substantial benefit to these patients. Some studies have reported that LA dilatation is an independent predictor of late TR recurrence during the follow-up period [19]. However, no investigators have focused on the value of LA strain. In the present study, LA reservoir strain was predictive of recurrence and had moderately high sensitivity and specificity. As we know, differing parameters regarding predictors of recurrence may reflect different causes of functional TR. LA reservoir function represents LA filling by pulmonary vein flow during ventricular systole and demonstrates stiffness of the left atrium, producing higher pressures. For example, LA reservoir strain has previously been correlated with left ventricular filling parameters and negatively correlated with mean pulmonary artery pressure and pulmonary capillary wedge pressure [20, 21]. Owing to the pathophysiological link between left-side heart diseases and the development of functional TR, the finding that recurrent TR could be predicted by parameters correlating with LA function can be explained. In the meantime, it is noteworthy that the damage to atrial function caused by the stenotic mitral orifice and active inflammatory lesions is already present before surgery. Even if a stenotic lesion is relieved, pulmonary vascular remodelling and elevated pulmonary vascular resistance may continue to occur and lead to morphological changes of the tricuspid valve and TR recurrence. Additionally, the relationship between LA reservoir strain and LAVI attracted our attention. In the present study, patients in Group II demonstrated lower value of atrial volume index in addition to lower reservoir atrial strain. The relationship is distinct from previous studies that have determined the inverse correlation between the two parameters [22]. This is probably because these patients have already developed a low flow state due to pulmonary vascular remodelling and irreversible obliterative changes. This pathophysiological change reduces congestive symptoms by decreasing venous return and leads to lower value of left atrial volume index, albeit at the expense of a reduction in cardiac output. However, further studies are needed to address this speculation. All these results suggest that surgeons should focus on correcting LA function disorders and reversing pulmonary vascular remodelling in rheumatic mitral stenosis patients undergoing MVR with concomitant TVA for functional TR to reduce recurrence effectively.

The present study also found that the preoperative mean transmitral gradient was an independent predictor of recurrence and had moderately high sensitivity and specificity. The mean transmitral gradient reflects changes in the degree of valve stenosis as well as LA compliance and cardiac output. A higher mean gradient, with an expected increase in PA pressure, could further change the tricuspid annular size and function. This is one possible reason to predict TR recurrence for the mean transmitral gradient. Jong-MinSong et al. studied 71 patients who suffered moderate-to-severe functional TR before percutaneous mitral valvuloplasty. The results indicated that functional TR could be reduced when the transmitral gradient was fully relieved, which also confirmed that changes in the transmitral pressure gradient could influence functional TR [23]. However, there are also conflicting reports indicating that the transmitral gradient (peak or mean) on follow-up echocardiographic assessment failed to predict recurrence of functional TR [24]. The surgical treatment of rheumatic mitral stenosis in our study differed from that in the previously mentioned studies. The long*-*term outcome of percutaneous mitral valvuloplasty may be influenced by the postoperative transmitral gradient. In contrast, the patients in our study underwent MVR surgery, and the postoperative transmitral gradient was within a reasonable and consistent range. The results may highlight the prognostic significance of the preoperative transmitral gradient. However, the usefulness of this information in the real world is still unknown because the choice of surgical procedure for patients could be affected by many influencing factors.

In the current study, the preoperative PASP was similar between the successful repair and recurrent groups and atrial fibrillation also had no significant effect on recurrence. This may have occurred because most patients included in this study had pulmonary hypertension and atrial fibrillation and had a relatively severe condition. Thus, there was no significant difference between the two groups in this regard. Meanwhile, some studies have noted that the development of postoperative PASP is also a matter of concern in patients who have undergone TVA and other cardiac procedures [25, 26]. However, given that a clear postoperative TR Doppler spectrum could not be obtained in several patients, this aspect cannot be demonstrated in the present study and required further exploration in future studies. In fact, determining the predictors or factors associated with recurrent functional TR after repair is a complicated topic because the results will likely be related to the underlying disease. In this study, rheumatic mitral stenosis was the dominant disease. It is known to cause secondary pulmonary hypertension, RV dysfunction, and functional TR. However, whether the findings could be applied to more routine forms of functional TR such as ischaemic cardiomyopathy or nonischaemic cardiomyopathy, where intrinsic RV dysfunction is greater, remains uncertain. Therefore, this is an important direction for future research to address.

Our study has several limitations. First, this study was a single-centre study, and the small cohort size and selection bias reduces the possibility of extensive analysis and subgroup comparisons. These results should be interpreted with caution. Moreover, follow-up was performed at the discretion of the physician caring for the patient and we did not explore effect of LA strain on long-term survival and predictors of complications and mortality. Thus, the conclusion of this study still requires verification in large, prospective, randomized studies. Second, we cannot guarantee that TR was functional and not related to rheumatic involvement of the tricuspid valve in all cases. However, during the last echocardiographic follow-up, an experienced ultrasound operator did not find direct evidence of rheumatic involvement of the tricuspid valve, indicating that the results were credible. Third, different TVA techniques may affect patients’ outcomes. However, in the present study, we found that there was no difference in TVA technique between the two groups. The improvement of surgical skills and similar state of disease could partly explain this result. Thus, we suggest that the TVA technique applied did not greatly affect the overall results and that a longer follow-up time is needed. Finally, the dependability of the LA strain is affected by operators and image quality and consumes more time for determination than conventional echocardiographic parameters. However, several studies have confirmed its reliability and provided normal reference values [27], making the LA strain a reliable parameter to use in future clinical practice.

## Conclusion

LA strain was correlated with preoperative functional TR severity in patients with rheumatic mitral stenosis. In the multivariate analysis, the LA reservoir strain and preoperative mean transmitral gradient emerged as independent predictive factors for recurrent TR after TVA during long-term follow-up. The present study also noted that surgeons should focus on correcting LA function disorders and reversing pulmonary vascular remodelling to reduce TR recurrence for such patients.

## Supplementary Information

**Additional file 1.**

## Data Availability

The datasets generated and analysed during the current study are not publicly available because they contain information that could compromise the privacy of research participants; however, the data are available from the first author (mql90937@163.com) on reasonable request.

## Abbreviations

TR: Tricuspid regurgitation
TVA: Tricuspid valve annuloplasty
LA: Left atrium
RV: Right ventricle
MVR: Mitral valve replacement
TTE: Transthoracic echocardiography
MVA: Mitral valve area
2D: 2-dimensional
PHT: Pressure half-time
TAD: Tricuspid annular diameter
PASP: Pulmonary artery systolic pressure
LAVI: Left atrial maximal volume index
LASV: Left atrial stroke volume
LAEF: Left atrial emptying fraction
LAEI: Left atrial expansion index
LVEF: Left ventricular ejection fraction
2D-STE: Two-dimensional speckle-tracking echocardiography
ROC: Receiver operating characteristic curve
AUC: Area under the receiver operating characteristic curve
NYHA: New York Heart Association
LVEF: Left ventricular ejection fraction
LAS-r: Left atrial reservoir strain
LAS-cd: Left atrial conduit strain
LV-PGLS: Left ventricular peak global longitudinal strain
CI: Confidence interval
