# Supplementary material for: The role of left atrial strain in patients with functional tricuspid regurgitation before and after annuloplasty: a long-term follow-up study
Source: Cardiovasc Ultrasound. 2021 Oct 18;19:33. doi: 10.1186/s12947-021-00264-z (PMC8522237; doi:10.1186/s12947-021-00264-z)
Supplement: Supplementary file 1 — Additional file 1. [file 12947_2021_264_MOESM1_ESM.docx]

Supplementary Material Table S1 Adjusted logistic regression analysis for predicting the recurrence of TR after TVA

| Variables | Logistic regression analysis | β | 95% CI | *p* value |
| --- | --- | --- | --- | --- |
| Heart rate (beats/min) | Univariate analysis | 1.04 | 1.01-1.07 | 0.01^*^ |
|  | Adjusted for TR severity | 1.04 | 0.00-1.07 | 0.09 |
|  | Adjusted for TR severity and TA maximal diameter | 1.04 | 0.00-1.07 | 0.10 |
|  | Adjusted for TR severity and TA minimal diameter | 1.04 | 0.00-1.07 | 0.09 |
| Atrial fibrillation | Univariate analysis | 0.44 | 0.09-2.23 | 0.32 |
|  | Adjusted for TR severity | 0.63 | 0.11-3.62 | 0.61 |
|  | Adjusted for TR severity and TA maximal diameter | 0.59 | 0.10-3.45 | 0.56 |
|  | Adjusted for TR severity and TA minimal diameter | 0.64 | 0.14-2.86 | 0.55 |
| MVA by 2D planimetry (cm^2^) | Univariate analysis | 0.07 | 0.01-0.59 | 0.07 |
|  | Adjusted for TR severity | 0.08 | 0.01-0.77 | 0.09 |
|  | Adjusted for TR severity and TA maximal diameter | 0.09 | 0.01-0.79 | 0.09 |
|  | Adjusted for TR severity and TA minimal diameter | 0.08 | 0.01-0.77 | 0.09 |
| MV mean gradient (mm Hg) | Univariate analysis | 1.19 | 1.04-1.35 | 0.01^*^ |
|  | Adjusted for TR severity | 1.15 | 1.01-1.32 | 0.04^*^ |
|  | Adjusted for TR severity and TA maximal diameter | 1.16 | 1.01-1.33 | 0.04^*^ |
|  | Adjusted for TR severity and TA minimal diameter | 1.15 | 1.01-1.33 | 0.04^*^ |
| PASP (mm Hg) | Univariate analysis | 1.02 | 0.99-1.05 | 0.14 |
|  | Adjusted for TR severity | 1.01 | 0.98-1.05 | 0.46 |
|  | Adjusted for TR severity and TA maximal diameter | 1.01 | 0.98-1.05 | 0.48 |
|  | Adjusted for TR severity and TA minimal diameter | 1.01 | 0.98-1.05 | 0.45 |
| LV end-diastolic volume (mL) | Univariate analysis | 0.98 | 0.97-1.01 | 0.05 |
|  | Adjusted for TR severity | 0.98 | 0.95-1.00 | 0.07 |
|  | Adjusted for TR severity and TA maximal diameter | 0.98 | 0.95-1.00 | 0.09 |
|  | Adjusted for TR severity and TA minimal diameter | 0.98 | 0.95-1.00 | 0.08 |
| LV end-systolic volume (mL) | Univariate analysis | 0.96 | 0.93-1.01 | 0.05 |
|  | Adjusted for TR severity | 0.96 | 0.92-1.01 | 0.10 |
|  | Adjusted for TR severity and TA maximal diameter | 0.97 | 0.92-1.01 | 0.12 |
|  | Adjusted for TR severity and TA minimal diameter | 0.96 | 0.92-1.01 | 0.11 |
| LA maximal volume (mL) | Univariate analysis | 0.99 | 0.99-1.01 | 0.18 |
|  | Adjusted for TR severity | 0.99 | 0.99-1.00 | 0.07 |
|  | Adjusted for TR severity and TA maximal diameter | 0.99 | 0.99-1.00 | 0.08 |
|  | Adjusted for TR severity and TA minimal diameter | 0.99 | 0.99-1.00 | 0.07 |
| LA minimal volume (mL) | Univariate analysis | 0.99 | 0.99-1.01 | 0.18 |
|  | Adjusted for TR severity | 0.99 | 0.98-1.00 | 0.06 |
|  | Adjusted for TR severity and TA maximal diameter | 0.99 | 0.98-1.00 | 0.06 |
|  | Adjusted for TR severity and TA minimal diameter | 0.99 | 0.98-1.00 | 0.06 |
| LAVI (mL/m^2^) | Univariate analysis | 0.99 | 0.99-1.01 | 0.18 |
|  | Adjusted for TR severity | 0.99 | 0.97-1.00 | 0.06 |
|  | Adjusted for TR severity and TA maximal diameter | 0.99 | 0.98-1.00 | 0.07 |
|  | Adjusted for TR severity and TA minimal diameter | 0.99 | 0.97-1.00 | 0.06 |
| LAS-r (%) | Univariate analysis | 0.77 | 0.63-0.93 | 0.01^*^ |
|  | Adjusted for TR severity | 0.79 | 0.65-0.97 | 0.02^*^ |
|  | Adjusted for TR severity and TA maximal diameter | 0.79 | 0.65-0.97 | 0.02^*^ |
|  | Adjusted for TR severity and TA minimal diameter | 0.79 | 0.64-0.97 | 0.02^*^ |
| LAS-cd (%) | Univariate analysis | 1.26 | 1.03-1.55 | 0.07 |
|  | Adjusted for TR severity | 1.24 | 1.01-1.53 | 0.04^*^ |
|  | Adjusted for TR severity and TA maximal diameter | 1.24 | 1.01-1.53 | 0.05 |
|  | Adjusted for TR severity and TA minimal diameter | 1.24 | 1.01-1.53 | 0.05 |

CI Confidence interval; TR Tricuspid regurgitation; TA Tricuspid annulus; MVA Mitral valve area; 2D Two dimension; MV Mitral valve; PASP Pulmonary arterial systolic pressure; LV Left ventricle; LA Left atrium; LAVI Left atrial maximal volume index; LAS-r Left atrial reservoir strain; LAS-cd Left atrial conduit strain.

^*^P<0.05 for logistic regression.
